# Factors associated with treatment-seeking for malaria in urban poor communities in Accra, Ghana

**DOI:** 10.1186/s12936-018-2311-8

**Published:** 2018-04-16

**Authors:** Raphael Baffour Awuah, Paapa Yaw Asante, Lionel Sakyi, Adriana A. E. Biney, Mawuli Komla Kushitor, Francis Agyei, Ama de-Graft Aikins

**Affiliations:** 1 Regional Institute for Population Studies, University of Ghana, Accra, Ghana; 2 Department of Psychology, University of Ghana, Accra, Ghana; 3 Centre for Migration Studies, University of Ghana, Accra, Ghana

**Keywords:** Malaria, Treatment seeking, Urban poor communities, Accra, Ghana

## Abstract

**Background:**

In Ghana, about 3.5 million cases of malaria are recorded each year. Urban poor residents particularly have a higher risk of malaria mainly due to poor housing, low socio-economic status and poor sanitation. Alternative treatment for malaria (mainly African traditional/herbal and/or self-medication) is further compounding efforts to control the incidence of malaria in urban poor communities. This study assesses factors associated with seeking alternative treatment as the first response to malaria, relative to orthodox treatment in three urban poor communities in Accra, Ghana.

**Methods:**

This cross-sectional study was conducted in three urban poor localities in Accra, Ghana among individuals in their reproductive ages (15–59 years for men and 15–49 years for women). The analytic sample for the study was 707. A multinomial regression model was used to assess individual, interpersonal and structural level factors associated with treatment-seeking for malaria.

**Results:**

Overall, 31% of the respondents sought orthodox treatment, 8% sought traditional/herbal treatment and 61% self-medicated as the first response to malaria. At the bivariate level, more males than females used traditional/herbal treatment and self-medicated for malaria. The results of the regression analysis showed that current health insurance status, perceived relative economic standing, level of social support, and locality of residence were associated with seeking alternative treatment for malaria relative to orthodox treatment.

**Conclusions:**

The findings show that many urban poor residents in Accra self-medicate as the first response to malaria. Additionally, individuals who were not enrolled in a health insurance scheme, those who perceived they had a low economic standing, those with a high level of social support, and locality of residence were significantly associated with the use of alternative treatment for malaria. Multi-level strategies should be employed to address the use of alternative forms of treatment for malaria within the context of urban poverty.

## Background

Malaria is a major public health challenge in many low- and middle-income countries (LMICs). The increase in morbidity and mortality from malaria in LMICs is partly due to under-resourced health systems and behavioural factors [1]. Sub-Saharan Africa has the highest rate of morbidity and mortality from malaria. Estimates in 2015 showed that about 90 and 92% of the global proportion of malaria cases and deaths, respectively, were recorded in the region [2].

In Ghana, it is estimated that 3.5 million cases of malaria are recorded each year [3]. Evidence shows that malaria contributes to about 38% of all outpatient cases, and almost 50% of under-five hospital admissions in the country [4]. Malaria has been considered a predominantly rural disease in Ghana and many African countries, mainly because suitable vector breeding sites are rare in highly populated areas [5, 6]. Although there is evidence to show that there is a reduction of mosquito-breeding sites with increasing proximity to the centre of urban areas [7], there are many recorded cases of malaria transmission in urban areas [8].

Ghana has experienced an increase in urbanization due to the rapid rise in the population. More than 50% of the population live in urban areas [5]. Rapid urbanization has also created a problem of urban poverty. Urban poor residents particularly have a higher risk of malaria than previously acknowledged [9, 10]. Poor housing, low socio-economic status and poor sanitation (leading to the development of breeding sites) in urban poor communities substantially increase the risk of malaria. This presents a challenge to the control of malaria in such settings.

A number of interventions over the last two decades have been rolled out to reduce the incidence of malaria in Ghana. These include the malaria action plan and the roll back malaria initiative [3]. Despite these interventions, the malaria incidence in Ghana is still high. It has been reported that the incidence of malaria in Ghana increased by 3.5% in the first quarter of 2016 [11]. This has been attributed mainly to poor drug treatment practices, contributing to the widespread resistance of falciparum malaria [12].

In Ghana, and other African countries, home-based remedies that involve over-the-counter and leftover drugs, as well as the use of herbs, are often the first treatment strategies for malaria, with individuals proceeding to a medical facility for treatment if the condition worsens [13–16]. It has been suggested that misconceptions about malaria treatment as well as easy accessibility and availability to alternative treatment account for the choice of alternative treatment for malaria [17]. Additionally, poor service delivery at health facilities compels people to seek alternative treatment for malaria [18].

Implications of alternative treatment as a first response to malaria include misdiagnosis, over-or under-medication, delayed medical treatment, and resistance to malaria medication [19–21]. As malaria continues to be a health concern in Ghana and particularly in urban poor settings, studies that examine treatment-seeking among at-risk groups are needed.

## Conceptual approach

This study employs the use of the socio-ecological model. The model suggests that individual health behaviour is often influenced by factors at multiple levels, which include the individual, interpersonal and institutional/structural levels [22]. Individual factors such as gender, age, economic status, and education have been observed to influence one’s decision to engage with particular health systems [23]. Studies have also shown that interpersonal factors, such as social networks and support that people rely on, influence access and use of particular health services [24, 25]. Structural factors relate to institutional mechanisms put in place to ensure healthcare access. For example, it has been found that the proximity of a health facility and access to health insurance are associated with the use of orthodox treatment for infectious disease [26].

Although there is evidence of treatment-seeking behaviour for malaria and other infectious diseases in Africa [27–29], very little is known about treatment-seeking behaviour for malaria in the urban poor context. This study seeks to assess individual, interpersonal and structural level factors that are associated with seeking alternative treatment (herbal/traditional and self-medication) as the first response to malaria symptoms relative to the use of orthodox treatment in three urban poor communities in Accra, Ghana. This assessment will help identify the important correlates of treatment-seeking practices among urban poor residents, and also make it possible to identify individuals at most risk of exhibiting injurious behaviour through their practices. The findings from this study may have implications for policy on malaria among the urban poor.

## Methods

### Study setting

The study was conducted in three communities; James Town, Ussher Town and Agbogbloshie. All three communities are located in Accra, the capital of Ghana. James Town and Ussher Town are neighbouring indigenous communities located at the heart of the Central Business District (CBD) of Accra, while Agbogbloshie is a migrant settlement which is located close to the CBD. The three communities are generally characterized by poor environmental and sanitary conditions, which have implications for the health of residents, especially their susceptibility to infectious diseases, such as malaria.

Although all three localities are considered urban poor, Agbogbloshie exhibits the most slum-like characteristics as most residents lack proximal access to formal healthcare systems, access to water, lack appropriate drainage systems and toilet facilities, and dwell in makeshift housing structures. Agbogbloshie’s layout is unplanned and crowded with untarred walkways and areas that flood when rains occur.

James Town and Ussher Town are characterized by a high population density, with most housing structures lacking proper toilet facilities. Most of the housing structures in both communities are old and sometimes dilapidated. However, James Town and Ussher Town have well planned features with concrete (but uncovered) drains and tarred roads. There is a major health facility; the Ussher Town Polyclinic, which serves both communities.

All three localities have conditions that are conducive to the breeding of mosquitoes. The swampy ditches that arise in Agbogbloshie after rains, serve as breeding grounds for mosquitoes. The concrete drains that are filled with waste water and garbage in James Town and Ussher Town generate areas for mosquito larvae to form. Residents in all three localities are vulnerable to contracting malaria because of the relative ease with which breeding grounds for mosquitoes form, as well as the high population density.

### Sampling design and sampling technique

The data were drawn from the third wave (most current wave) of the Urban Health and Poverty (UHP) project conducted by the Regional Institute for Population Studies (RIPS), University of Ghana (UG). The UHP project has collected three waves of data from the aforementioned communities. The sampling design followed a two-stage sampling technique similar to that used for the Ghana Demographic and Health Surveys. Enumeration areas (EAs) were randomly sampled proportionate to the population sizes of the three localities. This resulted in five EAs being sampled from Agbogbloshie, 8 from James Town and 16 from Ussher Town. Subsequently, all structures in the sampled EAs were numbered and a household listing exercise conducted. Households on the list were cumulated, and this constituted the sampling frame. Forty households were then systematically sampled from each of the 29 EAs, resulting in a total of 1160 sampled households, and from these, all eligible household members were targeted for interviews. Eligible study participants were individuals in their reproductive ages (between 15–59 years for men, and 15–49 years for women). A detailed description of the sampling procedure has been published elsewhere [30].

### Data collection

Data collection took place between September and October, 2013. The data were collected by trained field assistants, comprising mainly graduate students at RIPS, UG. Interviews were conducted primarily in Ga (the native language of the two indigenous communities of James Town and Ussher Town) in addition to Twi (another common local language in all three localities) and English. A total of 782 eligible men and women were interviewed using an interviewer-administered questionnaire. The analytic sample for this study was 707. The final sample was obtained following list-wise deletion of variables with missing cases.

### Data entry and management

The survey was administered using paper-based questionnaires. Field editors checked responses on the field to minimize missing data and other errors. Upon completion of the survey, the data entry personnel entered the information captured using the Census and Survey Processing (CSPro) software. To ensure reliable data entry, the data were double entered. A data manager then generated and checked the entered data.

### Ethical considerations

Ethical approval for the study was granted by the Noguchi Memorial Institute for Medical Research—Institutional Review Board in August 2013 (certified Protocol Number 105/12-13). Appropriate consenting procedures were carried out before the start of an interview. Individuals who agreed to take part in the study were interviewed.

### Measurements

The dependent variable was treatment-seeking behaviour for malaria. Participants were asked which treatment option they first used when they had malaria. In other words, what their first response to malaria (symptoms) was. Responses were categorized as orthodox treatment (treatment of malaria using the formal health care system, such as a public/government or private health facility); herbal/traditional treatment (the use of herbs, either purchased or prepared); and, self-medication (which includes using shop-bought drugs or the use of drug peddlers).

The explanatory variables were gender, age, educational status (ever attended school or not), social support (how likely an individual will receive/ask for help with regard to medication, on a scale of 1–4), current health insurance status, perceived relative economic standing (how participants perceived their economic status relative to others in the community, on a scale of 1–5), frequency of suffering from malaria in the last 5 years, and locality of residence (Agbogbloshie, James Town, Ussher Town).

### Data analysis

Univariate results were expressed as means ± standard deviations (for continuous variables) and as percentages (for categorical variables). A cross tabulation was conducted between treatment seeking for malaria and gender, to examine gender differentials. A multinomial logistic regression analysis was used to assess the factors associated with seeking herbal/traditional treatment and self-medication for malaria relative to orthodox treatment as the first illness response. Selection of the variables was informed by the socio-ecological model and availability in the dataset. Data were analysed using IBM SPSS Statistics (version 23).

## Results

The characteristics of the study participants are presented in Table 1. More than half of the participants were females (55%). Majority of the participants were young adults (37%), and more than 90% had attended school. Relating to health insurance, about 6 out every 10 participants indicated that they were not currently enrolled on any health insurance scheme. The mean perceived relative economic standing and the mean level of social support were 2.6 and 2.2, respectively. The mean number of times that individuals had malaria in the last 5 years was 2.9.

**Table 1:** Descriptive statistics

Variables (SD)	n (%)/mean
Gender	
Male	315 (44.6%)
Female	392 (55.4%)
Age groups	
Young adults	260 (36.8%)
Middle-aged adults	216 (30.6%)
Older adults	231 (32.7%)
Ever attended school	
Yes	664 (93.9%)
No	43 (6.1%)
Health insurance status	
Currently enrolled	289 (40.9%)
Not currently enrolled	418 (59.1%)
Locality	
Agbogbloshie	98 (13.9%)
James Town	198 (28.0%)
Ussher Town	411 (58.1%)
Perceived relative economic standing	2.63 (± 0.96)
Level of social support	2.22 (± 1.16)
Frequency of malaria in last 5 years	2.92 (± 3.66)

Results are expressed as n (%) or mean (± SD)

**Fig. 1:**
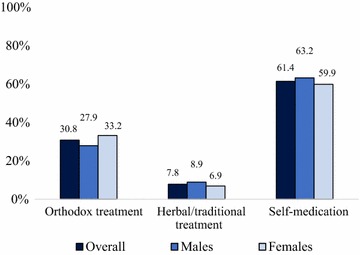
Treatment seeking for malaria by sex

Overall, 31% sought orthodox treatment as their first response to malaria. About 8% sought traditional/herbal treatment, while three out of every five individuals (61%) self-medicated as their first response to malaria. More males than females sought herbal/traditional treatment as the first response to malaria (8.9% compared to 6.9%). Additionally, more males self-medicated for malaria (approximately 63%) compared to females (60%) (Fig. 1).

Table 2 shows the factors associated with using herbal/traditional treatment and self-medication for malaria relative to the use of orthodox treatment. The model shows that one’s health insurance status and locality of residence (structural factors), level of social support (interpersonal factor) and perceived relative economic standing (individual factor) were significantly associated with other forms of treatment for malaria relative to orthodox treatment. Specifically, the findings show that individuals currently enrolled in a health insurance scheme were less likely to use herbal/traditional treatment, and also less likely to self-medicate for malaria relative to orthodox treatment (OR = 0.50, p < 0.01 and 0.48, p < 0.001, respectively). In relation to locality of residence, the findings show that compared to Ussher Town, individuals living in Agbogbloshie and James Town were more likely to use herbal/traditional treatment as the first response to malaria relative to orthodox treatment (OR = 7.47, p < 0.01 and 2.33, p < 0.001, respectively). Additionally, those in Agbogbloshie were more likely to self-medicate for malaria relative to using orthodox treatment (OR = 3.02, p < 0.01). A higher perception of economic standing compared to others was associated with lower odds of using herbal/traditional treatment as the first response to malaria relative to the use of orthodox treatment (OR = 0.72, p < 0.05). Also, an increase in an individual’s level of social support was associated with an increase in the odds of self-medication for malaria relative to using orthodox treatment (OR = 1.19, p < 0.01).

**Table 2:** Multinomial logistic regression for treatment seeking for malaria

Factors	Treatment seeking for malaria	
	Herbal/traditional treatment Odds ratio (95% CI)	Self-medication Odds ratio (95% CI)
Gender		
Male	1.40 (0.75–2.64)	1.18 (0.83–1.66)
Female *(RC)*	–	–
Age group		
Younger adults	0.70 (0.34–1.51)	1.15 (0.76–1.72)
Middle aged adults	1.32 (0.63–2.79)	1.44 (0.93–2.22)
Older adults *(RC)*	–	–
Ever attended school		
Yes	1.83 (0.48–6.97)	1.31 (0.64–2.68)
No *(RC)*	–	–
Health insurance status		
Currently enrolled	0.50 (0.26–0.95)**	0.48 (0.34–0.68)***
Not currently enrolled		
Locality		
Agbogbloshie	7.47 (2.89–19.31)***	3.02 (1.53–5.98)**
James Town	2.33 (1.16–4.67)**	0.90 (0.62–1.32)
Ussher Town *(RC)*	–	–
Perceived relative economic status	0.72 (0.50–1.00)*	0.90 (0.75–1.08)
Number of times with malaria in last 5 years	0.97 (0.87–1.08)	1.03 (0.98–1.08)
Social support	1.14 (0.75–2.61)	1.19 (1.02–1.38)**

RC is the reference category. * p < 0.10, ** p < 0.05, *** p < 0.001. The reference category for the dependent variable is orthodox treatment

Nagelkerke R^2^ value = 11.6%

## Discussion

The study sought to assess treatment-seeking in relation to malaria among residents of three urban poor localities in Accra. The findings show that majority of the study participants self-medicated for malaria. Additionally, the use of herbal/traditional treatment and self-medication relative to orthodox treatment were significantly predicted by two structural level factors (current health insurance status and locality of residence), one interpersonal level factor (level of social support) and one individual level factor (perceived relative economic standing).

The high proportion of individuals who self-medicated as first option for malaria treatment in this study is consistent with findings in previous studies in Ghana and other parts of Africa [31, 32]. In Ghana, access to drugs from pharmaceuticals and licensed chemical sellers without proper diagnoses and authorization, consumption of leftover drugs, and the use of home remedies are the main sources of self-medication for malaria [33]. It has been suggested that the high morbidity and mortality that characterizes malaria in Ghana and many countries in sub-Saharan Africa may be partly explained by the persistence and extent of self-medication [34]. Additionally, self-medication could potentially lead to complications due to over- and under-dosage of drugs, and delay in proper medical attention [35–37].

The findings further showed that being currently enrolled on health insurance is associated with lower odds of using herbal/traditional treatment and self-medication for malaria. Having active health insurance grants access to orthodox treatment since malaria is covered under the current National Health Insurance Scheme (NHIS) in Ghana [38]. Such individuals are therefore able to access the range of services for malaria treatment, which includes malaria diagnosis and access to recommended and effective medication. This finding indicates that health insurance provides financial protection for malaria treatment among urban poor residents. It also suggests that those who use orthodox treatment for malaria as a first response may ensure their continued enrolled status on the NHIS.

The study also found a negative association between perceived relative economic standing (a proxy measure for economic status) and the use of herbal/traditional treatment as the first response to malaria in the three urban poor communities. This finding establishes the relationship between economic status and treatment-seeking; that is, the lower an individual’s economic status, the higher the chance of using alternative treatment for malaria and vice versa. Financial inaccessibility has been identified as a major barrier to orthodox treatment for malaria [32, 39]. A positive association was observed between social support (for medication use) and self-medication for malaria. In other words, the higher the level of social support, the more likely an individual would self-medicate for malaria relative to using orthodox treatment. This finding indicates that this positive type of social support can negatively influence health-seeking behaviour [24]. In the three communities, there are widespread misconceptions about orthodox treatment for malaria and other conditions [40, 41]. As such, individuals who receive or ask for help for medication may be advised to forego orthodox treatment for alternative treatment, which is less costly. In a previous study in the three communities, poverty undermined treatment adherence for diabetes in a number of ways, including limiting access and use of orthodox medicines [42]. The finding in this study implies that financial constraints may result in the use of alternative treatment for malaria. Additionally, individuals who depend on others to finance their healthcare needs may be compelled to use cheaper forms of treatment, including self-medication, because the financial support they receive may be inadequate. This suggests that for the very poor, access to social protection, such as grants from Ghana’s flagship Livelihood Empowerment Against Poverty (LEAP) programme, should be an important resource alongside NHIS [43]. Qualitative studies should be conducted to further explore these social dynamics.

Of the three communities, participants living in Agbogbloshie were more likely to use herbal/traditional treatment and self-medicate compared to those living in Ussher Town. This could be attributed to a number of reasons. Residents in Agbogbloshie may not be adequately resourced to access orthodox treatment outside of their community, and as a result are likely to opt for herbal/traditional treatment or self-medication, which are more easily accessible, convenient and affordable. This suggests more structural barriers to health care for Agbogbloshie residents compared to residents of Ussher Town who have a major health facility. The location of a polyclinic in Ussher Town could be the reason its residents are more likely to seek biomedical treatment for malaria. Second, perceived effectiveness of alternative health systems, and beliefs on illness causation may account for the use of alternative forms of treatment for malaria in Agbogbloshie. Additionally, it is plausible that the choice of herbal/traditional treatment and self-medication for malaria in Agbogbloshie may be influenced by common health practices associated with many diseases that have existed in the community for a long period of time. On the other hand, those in James Town, compared to individuals living in Ussher Town, were more likely to use herbal/traditional treatment for malaria relative to orthodox treatment. Although James Town has improved access to health care, much like Ussher Town, anecdotal evidence suggests that the dominance of poorly regulated drug outlets, such as chemical shops and itinerant drug peddlers in many parts of the community, makes it easier for people to have access to herbal/traditional medication for malaria without proper authorization [40].

The finding that more males than females opt for traditional/herbal treatment and self-medication is similar to findings in another study conducted in the three communities [41]. In the study communities, men are unlikely to seek treatment if unwell, or when they seek treatment, will opt for alternatives outside the formal health care system due to community perceptions/myths regarding illness disclosure.

In relation to the socio-ecological model adopted for this study, the findings show the specific individual, interpersonal and structural factors that are associated with the use of alternative treatment for malaria relative to the use of orthodox treatment. These findings suggest that in the urban poor context in Ghana, it is important to focus on the specific factors that influence health behaviour. Psychological models of treatment-seeking behaviour must be adapted to intervention strategies, alongside socio-cultural and health economics models [42, 44]. At a general level, better public health education on malaria treatment is needed to address community and individual misconceptions about malaria treatment. At a specific level, one factor that cuts across all the levels of analysis is the financial factor. This is a cross-cutting issue in terms of the financial status of the individual with malaria, the level of financial support they receive from benefactors and the financial inaccessibility of orthodox treatment. As a result, health policy responses (such as access to NHIS) must be backed by responses from social welfare policies (such as the LEAP programme) to address the needs of poor individuals and families.

## Limitations

The study had a few limitations. First, recall bias may have compromised responses about participants’ first treatment action for malaria, and also responses to the question on frequency of malaria. Malaria was described by the interviewers to respondents who did not know or understand what the condition was, in order to capture responses that accurately reflected awareness/knowledge of the condition. However, it is likely that some respondents may have equated malaria with other types of fever. Also, the cross-sectional nature of the study limits the ability to infer causality. As such, the study solely examined associations between individual, social and structural factors and treatment-seeking. To fully understand health-seeking behaviour in the urban poor context, future studies should assess the history of treatment-seeking for a range of infectious diseases, and also use longitudinal data to track the use of health services. Additionally, qualitative studies can provide in-depth and nuanced information on health and treatment-seeking choices in the study communities.

## Conclusions

Generally, health-seeking behaviour in urban poor communities in Accra is pluralistic. Several treatment options exist and operate independently of the formal health care system. Unlike the formal health care system, which is subject to regulations, alternative treatment for various health conditions is largely unregulated. Alternative treatment options for malaria have been linked with poor malaria control, and may undermine malaria control efforts in urban poor communities. To avoid this situation from further escalating, health stakeholders should pay attention to the multilevel factors that are associated with seeking alternative treatment for malaria. Key factors to consider are economic status (individual level), social support (interpersonal level), health insurance status, and locality of residence (structural level). At these various levels of factors associated with the use of alternative treatment for malaria, the lack of financial resource in seeking orthodox treatment is clear. This should be taken into account when developing multilevel malaria treatment interventions within the context of urban poverty.
